# Friendship Diversity and Adolescents’ Civic Action and Commitment to Social Change

**DOI:** 10.3390/bs16091501

**Published:** 2026-08-27

**Authors:** Kara Kogachi, Sandra Graham

**Affiliations:** 1 Department of Child Development, California State University, Dominguez Hills, Carson, CA 90747, USA; kkogachi@csudh.edu; 2 Department of Education, University of California, Los Angeles, CA 90095, USA

**Keywords:** racial and ethnic diversity, adolescent friendships, civic engagement, collective action, commitment to social change, awareness of inequality, valuing diversity

## Abstract

This study examined whether racial and ethnic friendship diversity predicted adolescents’ civic action and commitment to social change, and the pathways leading to these outcomes. Using 2 years of data from a large, diverse sample of high school students (N = 3322; 47% female; Mage at T1 = 17.53; 11% Black/African American, 16% East/Southeast Asian, 36% Latiné, 24% White, 12% Multiracial/Biracial), findings revealed that friendship diversity at T1 (11th grade) predicted higher civic action and commitment 1 year later at T2 (12th grade). Across two mediation models, these associations operated indirectly through greater awareness of inequality and more positive beliefs about the value of diversity. Direct effects were nonsignificant once mediators were included. Multigroup tests showed similar pathways across racial/ethnic groups. Findings highlight friendship diversity as a developmental context that predicted greater awareness of inequality and valuing of diversity, which in turn was associated with civic action and commitment to social change. Results underscore the importance of racially and ethnically diverse friendship networks and awareness of inequality and valuing diversity as pathways to promote adolescents’ civic action and commitment social change.

## 1. Introduction

As the school-age population in the U.S. becomes increasingly racially and ethnically diverse, understanding how to foster positive relations between adolescents of different racial and ethnic groups is critical. The benefits of intergroup contact on reducing prejudice and improving intergroup attitudes have been well-documented (Pettigrew & Tropp, 2006). However, less is known about whether positive intergroup contact, including cross-race and cross-ethnic friendships, is associated with other important intergroup outcomes, such as collective action and support for social change (for a review, see Cocco et al., 2024). In the social psychology literature using adult and college samples, evidence has been mixed (see Reimer & Sengupta, 2023, for a meta-analysis), with some studies finding that positive intergroup contact can inadvertently reduce perceptions of injustice and motivation to act (e.g., Saguy et al., 2009), whereas other studies find that it can increase support for social change and action (e.g., Árnadóttir et al., 2024; Tropp & Dehrone, 2023). Few studies have tested these links in adolescence, despite this being a developmental period when civic orientations are emerging and experiences with people from different backgrounds and perspectives shape how youth think about social issues (Flanagan & Levine, 2010).

Moreover, intergroup contact research often relies on binary distinctions (e.g., advantaged vs. disadvantaged) or measures of concentration (e.g., proportion of White friendships or minoritized friendships), which do not fully capture the complexity of networks spanning more than two racial/ethnic groups (Cocco et al., 2024). Therefore, the present study examined whether racial and ethnic friendship diversity predicts civic action (i.e., engagement in civic and political behaviors aimed at promoting social change or addressing inequality) and commitment to social change (i.e., valuing political and social involvement as a personal goal), and factors that explain these associations.

### 1.1. The Effect of Positive Intergroup Contact on Collective Action and Support for Social Change

Intergroup contact theory proposes that meaningful contact with members of other groups can improve intergroup relations (Allport, 1954). Cross-group friendships are especially important forms of contact because they often satisfy Allport’s optimal conditions, including cooperation, common goals, and relatively equal status over time and across settings (e.g., Davies et al., 2011). While positive intergroup contact can reduce prejudice and interpersonal discrimination, prejudice reduction alone is insufficient to address structural and institutional inequalities and discrimination. Rather, collective action efforts aimed at improving the conditions of disadvantaged group members such as signing petitions, taking part in strikes, volunteering, valuing political and social involvement, and supporting egalitarian and reparative policies, are needed to promote social change and greater equality in society. In the social psychology literature using adult and college student samples, evidence is mixed regarding whether positive contact facilitates or undermines social change, and effects differ for advantaged versus disadvantaged group members. For advantaged groups, past research findings indicate that friendships with disadvantaged groups can enhance majority members’ support for social change (Cocco et al., 2024). For example, among White college students, having more friendships with racial/ethnic minoritized peers was associated with greater awareness of racial injustice and involvement in action to help minorities (Carter et al., 2019), and increased support of reparative policies (Northcutt Bohmert & DeMaris, 2015).

For members of disadvantaged groups, some research suggests that intergroup friendships can undermine efforts to achieve social change and challenge the status quo by reducing perceptions of injustice (Dixon et al., 2010; Saguy et al., 2009) and diminishing perceived discrimination (Tropp et al., 2012). In one study, for example, for racial/ethnic minoritized college students, having a greater proportion of White friends was associated with decreased awareness of racial injustice and involvement in action efforts to help minoritized groups (Carter et al., 2019). Other work suggests that the effects of contact are context-dependent and not uniformly negative for disadvantaged groups. Positive contact can strengthen support for social change and willingness to work in solidarity when it supports perceptions of shared goals and mutual respect (e.g., Árnadóttir et al., 2024; Hässler et al., 2020). Yet few studies have investigated the effect of both intragroup and intergroup friendships. When both forms of contact are considered, evidence suggests that contact with ingroup members can motivate disadvantaged groups to support social change (e.g., Sengupta et al., 2015). However, little is known about how the broader racial and ethnic diversity of adolescents’ friendship networks relates to civic action and commitment to social change.

### 1.2. Racial and Ethnic Diversity of Adolescent Friendship Networks

Friendships are a central context for development during adolescence, and race and ethnicity remain defining features of adolescent friendship networks (Graham & Echols, 2018). Compared to childhood, friendships during adolescence are more likely to be based on psychological characteristics such as trust and self-disclosure (Berndt, 2002), providing greater opportunities for adolescents to understand peers’ perspectives and experiences. As the U.S. school-age population continues to diversify, understanding intergroup relations among different racial and ethnic groups is increasingly important (Jones & Dovidio, 2018; Tropp et al., 2012), making diverse adolescent friendship networks a particularly meaningful context for examining these processes. However, most developmental studies and intergroup contact research have operationalized friendships composition using concentration or binary distinctions (e.g., proportion cross-race/ethnic friendships; advantaged vs. disadvantaged) (Cocco et al., 2024; Hewstone, 2015). In intergroup contexts where there are more than two groups present, binaries can overlook whether cross-race/ethnic friends are from different racial/ethnic groups. Similarly, the focus on disadvantaged and advantaged groups overlooks groups who do not fit into this categorization and limits understanding of efforts for social justice, which often involve multiple groups (e.g., Dixon et al., 2020; Hässler et al., 2020). Despite the documented benefits of racial and ethnic diversity at the school and classroom level (Juvonen et al., 2018), few studies have systematically examined how exposure to diversity in adolescent friendship networks impacts adolescent outcomes with measures of exposure that capture the full range of diversity.

Emerging developmental research suggests that friendship diversity may prompt deeper engagement with questions of identity and social hierarchy. For example, racially and ethnically diverse friendship networks at the start of sixth grade have been associated with greater ethnic racial identity exploration (Rivas-Drake et al., 2017). Similar findings have been replicated with high school students (Lorenzo et al., 2024). Among White college students, Pauker et al. (2018) found that friendship diversity was associated with decreases in the endorsement of race essentialism, which was in turn related to increased cognitive flexibility and decreased modern racism and social dominance orientation (i.e., one’s degree of preference for inequality among social groups). This is consistent with the prejudice reduction perspective, which aims to change how individuals relate to other groups, and how individuals see themselves and their group in relation to outgroups through positive intergroup contact (Tropp & Dehrone, 2023). Thus, to the extent that adolescents have more racially and ethnically diverse friendships and greater exposure to a variety of different groups, the more opportunities there are for reconfiguring their construal of relations between groups.

In line with this work, we focus on awareness of inequality and valuing diversity as potential mediating processes through which racially and ethnically diverse friendships shape adolescents’ thinking about social groups. Exposure to diverse peers may highlight that groups differ in experience and treatment, while also reinforcing the value of engaging with people who are different. Developmental research supports this focus, showing that cross-race/ethnic friendships are associated with greater perspective-taking (Devine et al., 2024), which can facilitate adolescents’ developing understanding of patterns of inequality and difference across social groups. Based on sociopolitical development theory, adolescents’ recognition of systemic inequality reflects a form of social analysis that can motivate commitment and action (“societal involvement”), including community service, civic engagement, and sociopolitical activism (Watts & Flanagan, 2007). Related work on critical consciousness also suggests awareness of inequality is a precursor to civic action and efforts to address inequality (Diemer et al., 2021) and continues to develop across adolescence, including among racially and ethnically minoritized adolescents (e.g., Umaña-Taylor et al., 2014; Wray-Lake et al., 2023).

Exposure to diverse social networks defined as a mix of ingroup and outgroup members has also been associated with greater social cohesion and more positive beliefs about the value of diversity in adult samples (Kauff et al., 2021; Ramos et al., 2024). Valuing diversity has been shown to mediate links between intergroup contact and support for inclusive policies (e.g., Leslie et al., 2020; Wallrich et al., 2022). Together, this work suggests that racially and ethnically diverse friendships may shape adolescents’ civic action and commitment to social change through these mechanisms. However, these pathways have not been examined in adolescent friendship networks.

### 1.3. The Current Study

The present study capitalized on a large racially and ethnically diverse sample of late adolescents in high school (11th–12th grade) to examine how the racial and ethnic composition of adolescents’ friendship networks are related to civic action (i.e., engagement in civic and political behaviors aimed at promoting social change or addressing inequality) and commitment to social change (i.e., valuing political and social involvement as a personal goal). In doing so, we extend prior intergroup contact research on collective action and support for social change to adolescence and advance this literature by using a more comprehensive measure of intergroup contact based on the racial and ethnic diversity of adolescents’ friendship networks. Specifically, we first examined whether commonly used measures of friendship composition (i.e., the proportion of White and racially and ethnically minoritized friends) and friendship diversity predicted civic action and commitment to social change. We then examined whether awareness of inequality and valuing diversity mediated the associations between friendship diversity and adolescents’ later civic action and commitment to social change as these beliefs reflect adolescents’ developing understanding of social group differences that may link social experiences to civic outcomes. Given evidence that friendships may have different implications for social change among White and racially and ethnically minoritized adolescents, we also examined whether these associations varied by adolescents’ racial and ethnic background. It was hypothesized that greater racial and ethnic diversity in adolescents’ friendship networks would predict greater civic action and commitment to social change for all adolescents, and that these links would be mediated by awareness of inequality and valuing diversity.

## 2. Materials and Methods

### 2.1. Participants

The analytic sample included 3322 students from 142 high schools (47% female, Mage = 17.53 years) who were participating in a larger longitudinal study (Juvonen et al., 2018). Data were collected in the spring of 11th and 12th grade. Because friendship diversity and the proportion-based friendship measures were focal predictors, the analytic sample was limited to students with sufficient 11th-grade friendship network data to compute racial and ethnic friendship composition. Based on self-reported race and ethnicity, the analytic sample was racially and ethnically diverse (11% Black/African American, 12% multiethnic/biracial, 16% East/Southeast Asian, 24% White, and 36% Latiné). Regarding gender, 50.7% identified as cisgender female, 46.8% cisgender male, 1.8% nonbinary, and 0.7% gender questioning.

### 2.2. Procedure

Participants were drawn from a larger sample of 5.991 adolescents recruited in 6th grade from 26 racially and ethnically diverse middle schools in California in three cohorts. The three cohorts were surveyed each year in middle and high school. Parental consent and student assent were first obtained in 6th grade and then re-obtained in 9th grade. Prior to completing the questionnaires, students were informed about confidentiality and reminded that study participation was voluntary. Students completed electronic questionnaires in school on iPads during the spring of each high school year. Instructions for completing the survey were audio taped and all students worked at their own pace. After completing each survey, which took approximately 45–60 min in 11th and 12th grade, students received $35 cash or gift certificate compensation. The study was approved by the Institutional Review Board at UCLA (IRB protocol number 11-002066).

### 2.3. Measures

School Racial and Ethnic Diversity. Objective school racial and ethnic diversity was measured based on school-level race/ethnicity data collected from the California Department of Education (CDE) and using Simpson’s (1949) diversity index:

$$D_{S}=\;1\;-\;\sum_{i-1}^{g}{P_{i}}^{2}$$

where *P* is the proportion of students in the school who are in racial/ethnic group *i*. This proportion is squared (${P_{i}}^{2}$), summed across *g* groups, and then subtracted from 1. $D_{S}$ gives the probability that any two students randomly selected from a school will be from different racial/ethnic groups. Thus, Simpson’s index captures the number of different groups in a setting and the relative representation of each group. Values can range from 0 to approximately 1, where higher values indicate greater diversity (i.e., more groups that are relatively evenly represented). $D_{S}\;$ ranged from 0.04 to 0.78, (*M* = 0.53, *SD* = 0.19).

### 2.4. Predictors

Race and Ethnicity. Students self-reported their race and ethnicity selecting from 11 ethnic categories or could provide an open-ended answer if they identified as multi-ethnic or if their ethnicity did not fit any of the categories listed. The responses were later combined to fit larger categories (e.g., Black/African American and Black/other country of origin; East Asian and Southeast Asian; and Latino and Mexican/Mexican American). Due to the smaller sample sizes of other ethnic groups, only five of the largest ethnic groups were used in all analyses: Black/African American, East/Southeast Asian, Latiné, White, and Multiethnic/Biracial.

Racial and Ethnic Composition of Friendship Networks. A well-established sociometric procedure was used to measure friendships (Graham et al., 2014). In 11th grade (T1), students were asked to list the students in their grade who were their “good friends”. They were allowed to make an unlimited number of nominations. Unidirectional friendship nominations were used because who students considered to be their friend was of interest, regardless of whether the other person also viewed them as a friend. For each nominated friend, participants were then asked “What do you consider [friend’s name] ethnic group to be?” They were given the same 11 options used for self-reported ethnicity to select the monoracial/ethnic group or a multiethnic/biracial group to which they perceived each friend to belong. Measures of friendship composition were based on this subjective perception question. Friendship composition variables were computed only for students in schools with at least 60% valid friendship nomination data, and this threshold was applied to both friendship diversity and proportion-based friendship measures.

#### 2.4.1. Proportion White and Minoritized Friendships

The proportion of White friendships was calculated by dividing the number of White friends by the total number of friends. Mean proportions of White friends by participants’ race and ethnicity were as follows: White (*M* = 0.57, *SD* = 0.32, range = 0.00–1.00), Multiracial/Biracial (*M* = 0.29, *SD* = 0.30, range = 0.00–1.00), Asian (M = 0.13, SD = 0.22, range = 0.00–1.00), Latiné (*M* = 0.11, *SD* = 0.20, range = 0.00–1.00), and Black/African American (*M* = 0.09, *SD* = 0.18, range = 0.00–1.00).

#### 2.4.2. Proportion of Minoritized Friendships

Similarly, the proportion of minoritized (non-White) friendships was calculated by dividing the number of friends from racially/ethnically minoritized groups by the total number of friends. Mean proportions of minoritized friends by participants’ race and ethnicity were as follows: Black/African American (M = 0.90, SD = 0.19, range = 0.00–1.00), Latiné (*M* = 0.88, *SD* = 0.22, range = 0.00–1.00), Asian (*M* = 0.87, *SD* = 0.23, range = 0.00–1.00), Multiracial/Biracial (*M* = 0.69, *SD* = 0.30, range = 0.00–1.00), and White (*M* = 0.42, *SD* = 0.31, range = 0.00–1.00).

#### 2.4.3. Racial/Ethnic Diversity of Friendship Network

The racial/ethnic diversity of participant’s friendship networks was assessed using each friend’s race/ethnicity and computing Simpson’s diversity index, as described above. Mean diversity scores by participants’ race and ethnicity were as follows: Multiracial/Biracial (*M* = 0.49, *SD* = 0.25, range = 0.00–0.94), White (*M* = 0.41, *SD* = 0.25, range = 0.00–0.94), Latiné (*M* = 0.39, *SD* = 0.26, range = 0.00–0.94), Asian (*M* = 0.38, *SD* = 0.27, range = 0.00–0.88), and Black/African American (*M* = 0.34, *SD* = 0.29, range = 0.00–0.92).

### 2.5. Outcomes

Civic Action. Four items adapted from the civic participation subscale of the Active and Engagement Citizenship (AEC) questionnaire (Bobek et al., 2009) assessed how often adolescents engaged in civic and political activities aimed at promoting social change or addressing inequality over the past year (e.g., “how often have you participated in a community or political rally?”; “volunteered for a group that worked to reduce prejudice?”). Responses ranged from 1 (never) to 5 (more than once a month). Items were averaged, with higher scores indicating more frequent action (α = 0.86).

Commitment to Social Change. Six items adapted from the social agency subscale of the Cooperative Institutional Research Program (CIRP) Freshman Survey (CIRP, 2011) assessed the degree to which adolescents valued political and social involvement as a personal goal such as “working to stop prejudice” and “helping my community”. Items were rated using a 5-point scale from 1 (very important) to 5 (not at all important) and re-coded so that higher values indicated higher levels of commitment (α = 0.87).

### 2.6. Mediators

Awareness of Inequality. Four items were adapted from the CIRP (2011) Freshman (e.g., “Racial discrimination is no longer a major problem in America”) and rated on a 5-point scale (1 = strongly agree to 5 = strongly disagree) and reverse coded such that higher values indicate greater awareness of inequality (α = 0.76).

Valuing Diversity. Valuing diversity was measured using six items adapted from the Diversity Beliefs Scale (Wilton et al., 2020) (e.g., “ethnic diversity should be valued in society” and “it is important to recognize how each racial and ethnic group is unique”) and rated on a 5-point scale (1 = strongly agree to 5 = strongly disagree) and reverse coded such that higher values indicated greater valuing of diversity (α = 0.89).

### 2.7. Covariates

Covariates in addition to school racial and ethnic diversity were selected based on empirical associations with the key study variables and their potential to confound the associations of interest. Parent education and school proportion of students receiving free/reduced-price meals were included as individual- and school-level proxies for socioeconomic status because civic participation opportunities are unevenly distributed across schools and communities by socioeconomic status, and individuals from higher-SES backgrounds are more likely to participate in civic activities such as voting, campaigning, and volunteering (Levinson, 2010).

School Proportion Free or Reduced Priced Meals (FRPM). Proportion of students receiving FRPMs was included as a school-level covariate. FRPM served as a proxy for school SES and ranged from 0.02 to 0.96 (*M* = 0.45, *SD* = 0.23).

Parent Education. Parent educational attainment was used as a proxy for socioeconomic status. The parent or guardian who completed the informed consent at ninth grade self-reported their highest level of education on a 6-point scale (1 = elementary/junior high school, 2 = some high school, 3 = high school diploma or GED, 4 = some college, 5 = four-year college degree, and 6 = graduate degree) which ranged from ranged from 1 to 6 (*M* = 3.98, *SD* = 1.56).

Gender. Participants self-reported their gender by selecting from the following categories: boy/man, girl/woman, transgender, gender nonconforming, gender fluid, questioning/not sure, different identity, gender queer, or other. Responses were later recoded to fit the following four categories: cisgender girl (50.7%), cisgender boy (46.8%), nonbinary (1.8%), and questioning (0.7%).

Friendship Quality. For each nominated friend, participants responded to three items capturing support: “This friend helps me feel better when I’m upset”, “This friend sticks up for me/has my back”, “I can talk to this friend about planning for the future”. Responses to the three items ranged from 1 (no/hardly ever) to 3 (yes/almost all the time) and were averaged within and across all nominated friends (α = 0.84).

Analytic Plan.

The first aim of this study was to test the effects of friendships on key outcomes and mediators. A series of multilevel regression models were estimated in *M*plus 8.3. Friendship diversity (Simpson’s index) served as the primary predictor of civic action and commitment to social change (outcomes) as well as awareness of inequality and valuing diversity (mediators).

For the second aim, a series of mediation models in a structural equation modeling (SEM) framework were estimated to test direct and indirect effects with bias-corrected bootstrap confidence intervals (10,000 resamples) using the CLUSTER option to adjust standard errors for the nesting of students within schools. Model fit was evaluated using the adjusted *χ*^2^ test, comparative fit index (CFI; Bentler, 1990), and root mean squared error of approximation (RMSEA; Steiger, 1998). A nonsignificant *χ*^2^, a CFI above 0.90 (Bentler, 1990), and an RMSEA below 0.06 with relatively small standard errors (MacCallum et al., 1997) indicate the model adequately describes the relationships in the observed data. To test whether the strength of the mediation models differed across racial and ethnic groups, a series of multigroup SEMs were estimated to define group-specific bootstrap indirect effects and their contrasts using the MODEL CONSTRAINT command in *M*plus. This approach provides bias-corrected bootstrap confidence intervals, which are preferred for indirect effects because of their non-normal sampling distribution and provide more accurate control of Type I error and higher power compared to Wald tests (MacKinnon et al., 2004; Ryu, 2015).

For both aims, all models were estimated using full-information maximum likelihood (FIML) for missing data and included student-level covariates (gender, race/ethnicity, generational status, parent education, friendship quality) and school-level covariates (school racial and ethnic diversity, percent free/reduced-price meals). Missing data varied across study variables. Missingness was low for covariates, ranging from 0.0% to 6.9%. Missingness was higher for the 12th-grade outcomes, with 10.7% missing for civic action and 18.0% missing for commitment to social change. The mediator variables had the highest missingness, with 50.3% missing for both awareness of inequality and valuing diversity. Follow-up comparisons using chi-square tests and independent-samples *t*-tests to examine whether adolescents with and without mediator data differed on key study variables. Adolescents with and without mediator data did not differ on friendship diversity, proportion White friendships, proportion minoritized friendships, school diversity, or commitment to social change. Adolescents with valid mediator data reported slightly higher friendship quality (*M* = 2.75 vs. *M* = 2.69) and civic action (*M* = 1.73 vs. *M* = 1.66), slightly lower parent education (*M* = 3.94 vs. *M* = 4.18), and attended schools with slightly higher free/reduced-price lunch rates (*M* = 45.54 vs. *M* = 43.29). They were also more likely to be Latiné (39.5% vs. 27.4%) and third-generation (*M* = 42.5% vs. *M* = 32.2%), and less likely to be Asian (11.3% vs. 18.4%), second-generation (*M* = 49.1% vs. *M* = 55.0%), and cisgender female (*M* = 55.1% vs. *M* = 50.9%).

## 3. Results

### 3.1. Descriptive Statistics

Intercorrelations and descriptive statistics of study variables are included in Table 1. One-way analyses of variance (ANOVAs) indicated significant racial/ethnic group differences in the diversity of friendship networks, *F*(4, 3016) = 14.34, *p* < 0.001, the proportion of White friends, *F*(4, 3022) = 472.77, *p* < 0.001, the proportion of minoritized friends, *F*(4, 3016) = 445.07, *p* < 0.001, and school racial and ethnic diversity, *F*(4, 3261) = 15.93, *p* < 0.001. Post hoc Bonferroni analyses indicated that Multiracial/Biracial adolescents had significantly more diverse friendships (*M* = 0.49, *SD* = 0.25) than Black/African American (*M* = 0.34, *SD* = 0.29), Asian (*M* = 0.38, *SD* = 0.27), Latiné (*M* = 0.39, *SD* = 0.26), and White (*M* = 0.41, *SD* = 0.25) adolescents. Additionally, White and Latiné adolescents had more diverse friendships than Black/African American (*M* = 0.34, *SD* = 0.29) adolescents. White adolescents reported a significantly higher proportion of White friends (*M* = 0.57, *SD* = 0.32) than Black/African American (*M* = 0.09, *SD* = 0.18), Asian (*M* = 0.13, *SD* = 0.22), Latiné (*M* = 0.11, *SD* = 0.20), and Multiracial/Biracial adolescents (*M* = 0.29, *SD* = 0.30). Multiracial/Biracial adolescents also reported a significantly higher proportion of White friends than Black/African American, Asian, and Latiné adolescents.

Bonferroni post hoc tests also indicated that Black/African American (*M* = 0.90, *SD* = 0.19), Asian (*M* = 0.87, *SD* = 0.23), and Latiné adolescents (*M* = 0.88, *SD* = 0.22) reported significantly higher proportions of minoritized friends than Multiracial/Biracial (*M* = 0.69, *SD* = 0.30) and White adolescents (*M* = 0.42, *SD* = 0.31). Multiracial/Biracial adolescents also reported a significantly higher proportion of minoritized friends than White adolescents. Group differences in school racial and ethnic diversity were also found with Asian adolescents (*M* = 0.63, *SD* = 0.17) attending significantly more diverse schools than White (*M* = 0.55, *SD* = 0.15), Latiné (*M* = 0.52, *SD* = 0.14), and Multiracial/Biracial (*M* = 0.51, *SD* = 0.15) adolescents, and both Asian and White adolescents attending more diverse schools than Black/African American adolescents (*M* = 0.49, *SD* = 0.13).

### 3.2. Friendship Diversity

To address the first study aim, multiple regression analyses were conducted to test the effects of friendship diversity on the proposed outcome variables and mediators, controlling for all covariates. Results of the effects of racial and ethnic diversity of friendship networks, controlling for the proportion of White friendships, are depicted in Table 2. Findings revealed that friendship diversity predicted greater civic action (*b* = 0.12, *p* < 0.042) and commitment to social change (*b* = 0.16, *p* < 0.001). Friendship diversity also predicted greater awareness of inequality (*b* = 0.26, *p* < 0.004) and valuing diversity (*b* = 0.18, *p* < 0.001). These effects were not moderated by the friendship quality or race/ethnicity of participants. There were no significant effects of proportion of White friendships.

### 3.3. Proportion White and Minoritized Friendships

For comparison with prior research that has operationalized network composition using proportions, we also tested whether the proportion of White or minoritized friends predicted adolescents’ civic action, commitment to social change, and each of the mediators. As shown in Table 3 and Table 4, neither proportion of White or minoritized friendships significantly predicted civic action, commitment to social change, awareness of inequality, and valuing diversity. Furthermore, there were no significant interactions with adolescents’ race and ethnicity or friendship quality.

### 3.4. Direct and Indirect Effects of Friendship Diversity

**Figure 1:**
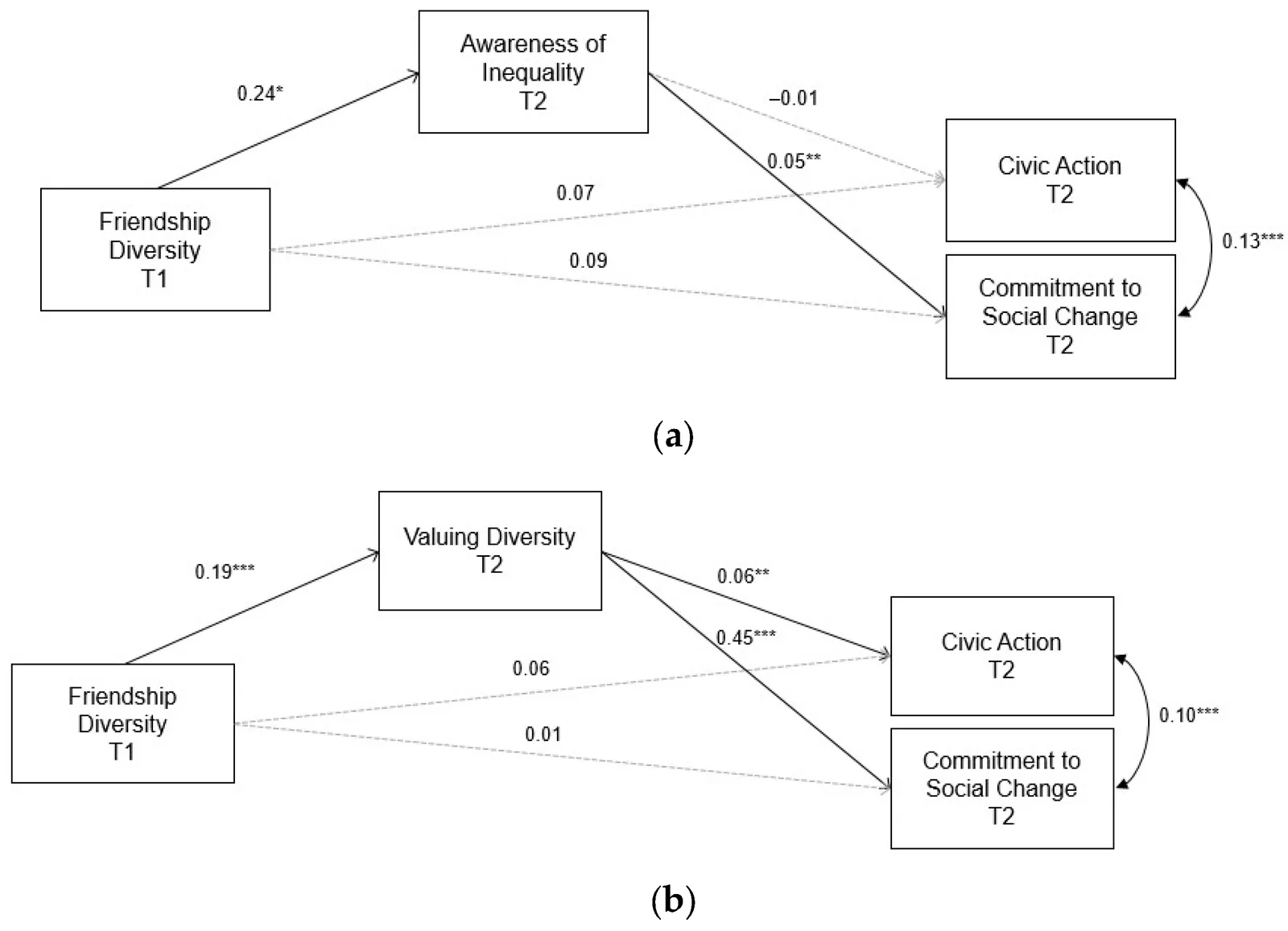
Unstandardized Parameter Estimates for the Mediation Model. (**a**) Awareness of Inequality; Model Fit: *χ*^2^(17) = 114.45, *p* < 0.001, RMSEA = 0.05, 90% CI [0.02, 0.06], CFI = 0.98. (**b**) Valuing Diversity; Model Fit: *χ*^2^(17) = 125.84, *p* < 0.001, RMSEA = 0.05, 90% CI [0.04, 0.06], CFI = 0.90. Note. * *p* < 0.05; ** *p* < 0.01; *** *p* < 0.001.

To address the second study aim, two separate structural equation models were estimated to test the direct and indirect effects of friendship diversity at T1 on each outcome at T2 through the two proposed mechanisms, controlling for all covariates (see Figure 1). All models used 10,000 bias-corrected bootstrap resamples to estimate indirect effects. An indirect effect coefficient with a 95% CI that did not include zero was considered to be significant. Multigroup models were then estimated to examine whether the direct and indirect effects differed by adolescents’ racial/ethnic group using Wald bootstrap tests.

#### 3.4.1. Pathways Through Awareness of Inequality

The awareness of inequality mediation model fit the data well: *χ*^2^(17) = 114.45, *p* < 0.001, RMSEA = 0.05, 90% CI [0.02, 0.06], CFI = 0.98 (see Figure 1). As hypothesized, friendship diversity predicted greater awareness of inequality (*b* = 0.24, *p* = 0.031). In turn, greater awareness of inequality was associated with greater commitment to social change (*b* = 0.05, *p* = 0.006), but was not significantly associated with civic action (*b* = −0.01, *p* = 0.47). Indirect effects indicated that friendship diversity predicted more commitment to social change, *b* = 0.01, 95% CI [0.00, 0.03], through higher awareness of inequality. The indirect effect for civic action was not significant, *b* = −0.01, 95% CI [−0.02, 0.00]. Direct effects of friendship diversity on both outcomes were nonsignificant once awareness of inequality was included in the model.

We next tested whether the main paths and indirect effects differed across racial and ethnic groups. Multigroup mediation models with bias-corrected bootstrapped indirect effects indicated no significant differences between any groups for the direct effects of friendship diversity on awareness of inequality and awareness of inequality on any outcomes, or for indirect effects predicting civic action or commitment to social change. Thus, the indirect pathways through awareness of inequality did not significantly differ across groups.

#### 3.4.2. Pathways Through Valuing Diversity

The valuing diversity mediation model showed adequate fit: *χ*^2^(17) = 125.84, *p* < 0.001, RMSEA = 0.05, 90% CI [0.04, 0.06], CFI = 0.90 (see Figure 1). As hypothesized, friendship diversity predicted greater valuing of diversity (*b* = 0.19, *p* < 0.001). In turn, valuing diversity was associated with greater civic action (*b* = 0.06, *p* = 0.004) and commitment to social change (*b* = 0.47, *p* < 0.001). Indirect effects indicated that friendship diversity predicted greater civic action, *b* = 0.02, 95% CI [0.01, 0.03], and commitment to social change, *b* = 0.09, 95% CI [0.03, 0.13], through valuing diversity. Direct effects of friendship diversity on both outcomes were nonsignificant once valuing diversity was included in the model.

Multigroup mediation models with bias-corrected bootstrapped indirect effects indicated no significant differences for the direct effects of friendship diversity on valuing diversity or for indirect effects predicting civic action or commitment to social change.

## 4. Discussion

The present findings support and extend prior intergroup contact research on collective action and support for social change. In our adolescent sample, friendship diversity, but not the commonly used proportion-based measures of friendship composition, was associated with greater civic action and commitment to social change through awareness of inequality and valuing diversity. By moving beyond proportion-based measures to capture the breadth of racial and ethnic groups represented in adolescents’ friendship networks, friendship diversity may better capture adolescents’ exposure to a broader range of racial and ethnic groups. The opportunities afforded by exposure to a broader range of racial and ethnic groups may be especially salient during adolescence, when friendships are more likely to be based on psychological characteristics such as trust and self-disclosure (Berndt, 2002), and civic orientations are also emerging (Flanagan & Levine, 2010). Together, these findings highlight friendship diversity as a salient developmental context for adolescents’ developing understanding of social group differences, reflected in greater awareness of inequality and valuing diversity, which in turn supported civic action and commitment to social change.

### 4.1. Racial and Ethnic Diversity of Friendship Networks and Its Mechanisms

In our adolescent sample, commonly used proportion-based measures (proportion White or minoritized friends) were not associated with adolescents’ civic action or commitment to social change. These null results suggest potential limitations of proportion-based measures, which capture binary distinctions (e.g., proportion White vs. non-White) and do not account for the heterogeneity of adolescents’ networks across multiple groups (Cocco et al., 2024; Dixon et al., 2020). By contrast, analyses of friendship diversity with a measure that captures the number and size of different groups represented, revealed consistent associations with adolescents’ civic action and commitment to social change, even after accounting for contextual factors such as school diversity and proxies for school SES (i.e., proportion students receiving free/reduced lunch). By modeling the breadth of groups represented, our findings suggest that friendship diversity may be a more meaningful measure for understanding adolescents’ civic action and commitment to social change. By modeling the breadth of racial and ethnic groups represented in adolescents’ friendship networks, our findings suggest that friendship diversity captures dimensions of intergroup contact not reflected in commonly used proportion-based measures. This broader exposure to different racial and ethnic groups may provide greater opportunities for adolescents to reconsider how they relate to other groups and understand their own group in relation to others through friendships (Tropp & Dehrone, 2023).

Friendship diversity was associated with adolescents’ civic action and commitment to social change through distinct pathways. As hypothesized, valuing diversity mediated the association between friendship diversity on both civic action and commitment to social change. Prior research has shown that valuing diversity mediates the association between intergroup contact and support for inclusive policies (e.g., Leslie et al., 2020; Wallrich et al., 2022). Extending this work to adolescence and to broader civic outcomes, the present findings suggest that valuing diversity may also help to explain how racially and ethnically diverse friendships are linked to both adolescents’ civic actions and their commitments to social change. Adolescents who viewed diversity as valuable were more likely to participate in civic and political activities aimed at promoting social change or reducing inequality, and were more committed to social change.

In contrast, awareness of inequality mediated the association between friendship diversity and commitment to social change, but not civic action, providing partial support for our hypothesis. Sociopolitical development theory posits that youths’ social analysis, including recognition that groups are treated unfairly, can motivate civic and political behaviors and commitments (Watts & Flanagan, 2007). Building on this theory, growing evidence suggests that awareness of inequality may relate differently to specific types of civic participation, with more consistent associations with political participation or justice-oriented activism than with service or prosocial forms of participation. For example, in a recent longitudinal study, adolescents’ awareness of societal inequality predicted increased political, but not prosocial, forms of civic engagement (Plummer et al., 2022). Similarly, Wegemer (2022) found that adolescents’ perceptions of inequities predicted activism (e.g., signing a petition, joining a protest), but not service behaviors (e.g., volunteering, organizing a fundraiser). In the present study, our civic action measure was adapted to capture participation in activities oriented toward social change or addressing inequality, even though these actions ranged from more explicitly political participation and activism (e.g., participating in a political rally) to service-oriented volunteering (e.g., joining a group working to reduce prejudice). Thus, the nonsignificant indirect effect through awareness of inequality may reflect that awareness is more proximally linked to political or justice-oriented activism than to service-oriented behaviors. These findings underscore the importance of distinguishing service-oriented participation from activism in future research.

As hypothesized, the pathways examined here functioned similarly across racial and ethnic groups. However, it is still possible that other processes linking diverse friendships and civic action and commitment to social change outcomes operate differently across groups. For White youth, friendships with racially and ethnically minoritized peers may encourage greater awareness of privilege and commitment to egalitarian values, particularly when these interactions involve open communication about group differences in power rather than focusing solely on commonalities (e.g., Tropp et al., 2021). For racial and ethnic minoritized youth, diverse friendships may foster both intragroup solidarity and intergroup coalition building, especially when they can connect around shared experiences of marginalization and discrimination (Carter et al., 2019) or fulfilling needs for empowerment and agency (Hässler et al., 2022). Thus, friendship diversity may also support a dual identity by both affirming ingroup membership and promoting a sense of belonging to the larger group, both of which have been shown to sustain motivation for social change (Dovidio et al., 2009; Glasford & Dovidio, 2011). Future work should continue to consider how the meaning and consequences of friendship diversity vary across status groups and contexts.

### 4.2. Limitations and Future Directions

While this study contributes to the friendship diversity literature, several limitations should be noted. First, the short-term longitudinal design limits causal inference and the ability to establish directionality. Guided by intergroup contact theory, we tested whether friendship diversity predicted outcomes and proposed pathways; however, these processes are likely reciprocal over time. Adolescents’ prior civic engagement and commitment to social change can shape both their friendship networks through selection processes and their thinking about group differences (awareness of inequality, valuing diversity). For example, Wegemer (2022) found that adolescents’ participation in service activities predicted friendship formation and selection. Longitudinal studies are needed to determine directionality and developmental trajectories. However, the timing of the study captures adolescents in the final years of high school, a period when civic orientations and plans for future engagement are becoming more salient (Flanagan & Levine, 2010). Second, friendship diversity was measured using perceived rather than friends’ self-reported race and ethnicity. Although this may introduce potential perceptual biases, adolescents’ perceptions are likely central to how they understand and navigate their social environments.

Third, although Simpson’s index captures structural diversity, it does not reveal the specific race or ethnicity of the friends or the content of friendships such as discussions about race or shared experiences of discrimination, which likely influence outcomes. Fourth, multigroup analyses revealed mostly nonsignificant contrasts as hypothesized; however, these results may reflect limited statistical power and should be interpreted with caution. Replication with larger and more diverse samples is needed to determine whether these patterns are robust. Finally, we focused on race and ethnicity; however, friendships are shaped by intersecting identities such as gender, class, and immigration status. Future research should examine how the intersection of multiple marginalized identities shapes adolescents’ capacity to recognize commonalities within their friendship networks and to engage in collective action that promotes justice for all (Terriquez et al., 2018). Multidimensional measures that also account for identities such as gender, class, and immigration status may reveal greater heterogeneity in friendship networks and stronger associations with civic action and commitment to social change. Future research could further examine the mechanisms underlying these associations, including the role of peer conversations and shared experiences within diverse friendship networks using qualitative approaches as well as influence and socialization processes using social network analysis.

## 5. Conclusions

Findings from this study revealed that the racial and ethnic diversity of adolescent friendship networks predicted adolescents’ civic action and commitment to social change. These links were explained by adolescents’ developing beliefs about social group difference, including greater awareness of inequality and valuing diversity. Rather than proportion measures that dichotomize groups, it was the diversity or breadth of friendship networks across different racial and ethnic groups that predicted greater awareness of social inequality and valuing diversity, which in turn was associated with greater civic action and commitment to social change.

The findings suggest that promoting friendships that bring together youth across racial and ethnic lines may not only reduce prejudice but also encourage civic action efforts and support for social change toward intergroup equality. This requires that schools themselves are diverse and inclusive by offering opportunities for meaningful intergroup contact and addressing the structural inequalities that shape intergroup relations (Graham, 2018). The mechanisms identified in this study also point to targets for intervention, particularly when diversity may not be possible. Schools can foster opportunities for adolescents to challenge group hierarchies, reflect on social and structural inequalities, highlight the value of diversity where minoritized groups’ voices can be centered and majority group members feel included. Curricula such as ethnic studies, for example, can foster critical reflection and sociopolitical development (e.g., Pinedo et al., 2025). At a time when ethnic studies programs are under increasing attack in this anti-diversity political climate (Rogers et al., 2022), they may be more important than ever for nurturing adolescents’ civic action and commitment to social change aimed at advancing a more just and inclusive society in an increasingly diverse world.

## Figures and Tables

**Table 1:** Intercorrelations and descriptive statistics of main study variables.

	1. Friendship Diversity	2. % White Friendships	3. % Minoritized Friendships	4. Friendship Quality	5. School diversity	6. % School Free/Reduced Lunch	7. Parent Education	8. Civic Action	9 Commitment to Social Change	10. Awareness of Inequality	11. Valuing Diversity
1	—										
2	−0.029	—									
3	−0.025	−0.977 **	—								
4	0.001	0.039 *	−0.030	—							
5	0.048 **	0.126 **	−0.124 **	0.033	—						
6	−0.182 **	−0.479 **	0.478 **	−0.016	−0.116 **	—					
7	0.100 **	0.350 **	−0.339 **	0.001	0.048 **	−0.365 **	—				
8	0.016	0.012	−0.004	0.078 **	−0.023	−0.022	0.049 **	—			
9	0.010	−0.057 **	0.061 **	0.168 **	−0.003	0.053 **	−0.017	0.162 **	—		
10	0.059 *	0.009	−0.008	0.024	−0.006	−0.017	0.046	−0.027	0.156 **	—	
11	0.065 *	−0.070 **	0.072 **	0.097 **	−0.024	−0.028	−0.010	0.050 *	0.371 **	0.225 **	—
Mean	0.401	0.244	0.742	2.719	0.525	45.230	3.980	1.685	3.981	3.986	4.279
*SD*	0.266	0.316	0.320	0.358	0.193	23.415	1.560	0.720	0.737	1.055	0.709
*N*	3322	3322	3322	3321	3246	3245	3092	2966	2725	1652	1652

*Note.* * *p* < 0.05; ** *p* < 0.01.

**Table 2:** Unstandardized coefficients from final multilevel models of friendship diversity predicting 12th grade civic action, commitment to social change, awareness of inequality, and valuing diversity.

	Civic Action	Commitment to Social Change	Awareness of Inequality	Valuing Diversity
	*b*(SE)	*b*(SE)	*b*(SE)	*b*(SE)
Intercept	0.941 (0.145) ***	2.838 (0.170) ***	3.245 (0.319) ***	3.536 (0.165) ***
*School-Level*				
School Diversity	−0.007 (0.078)	0.005 (0.075)	0.003 (0.138)	−0.053 (0.079)
% Free/Reduced Meals	0.000 (0.001)	0.001 (0.001)	−0.001 (0.001)	−0.002 (0.001) *
*Individual-Level*				
Gender				
Female (vs. Male)	0.068 (0.031) *	0.242 (0.031) ***	0.482 (0.059) ***	0.268 (0.032) ***
Non-binary (vs. Male)	−0.049 (0.098)	0.353 (0.113) **	0.414 (0.211)	0.264 (0.099) **
Questioning (vs. Male)	0.192 (0.166)	0.495 (0.097) ***	0.849 (0.139) ***	0.312 (0.140) *
Parent Education	0.018 (0.012)	0.002 (0.011)	0.025 (0.002)	−0.017 (0.012)
Generational Status				
Second Generation (vs. First)	−0.014 (0.058)	0.015 (0.055)	0.106 (0.111)	−0.108 (0.048) **
Third Generation (vs. First)	−0.041 (0.064)	−0.027 (0.061)	0.098 (0.117)	−0.183 (0.052) ***
Race/Ethnicity				
Black/African American (vs. White)	0.136 (0.060) *	0.306 (0.055) ***	−0.066 (0.118	0.123 (0.066)
East/Southeast Asian (vs. White)	0.101 (0.052) *	0.009 (0.057)	0.254 (0.104) *	0.150 (0.048) **
Latiné (vs. White)	0.001 (0.050)	0.093 (0.052	0.093 (0.086)	0.071 (0.049)
Multiethnic (vs. White)	0.021 (0.054)	0.091 (0.056)	0.210 (0.090)	0.100 (0.056)
Friendship Quality	0.197 (0.041) ***	0.292 (0.048) ***	0.068 (0.090)	0.261 (0.047) ***
Friend Racial/Ethnic Diversity	0.122 (0.060) *	0.164 (0.062) ***	0.287 (0.110) **	0.180 (0.069) ***
% White Friends	0.094 (0.063)	0.052 (0.069)	0.040 (0.117)	−0.034 (0.029)

Note. * *p* < 0.05; ** *p* < 0.01; *** *p* < 0.001.

**Table 3:** Unstandardized coefficients from final multilevel models of proportion White friendships predicting 12th grade civic action, commitment to social change, awareness of inequality, and valuing diversity.

	Civic Action	Commitment to Social Change	Awareness of Inequality	Valuing Diversity
	*b*(SE)	*b*(SE)	*b*(SE)	*b*(SE)
Intercept	1.049 (0.140) ***	2.911 (0.157) ***	3.644 (0.204) ***	3.110 (0.319) ***
*School-Level*				
School Diversity	−0.020 (0.076)	−0.012 (0.075)	−0.035 (0.091)	0.079 (0.139)
% Free/Reduced Meals	0.001 (0.001)	0.001 (0.001)	−0.002 (0.001) *	−0.001 (0.002)
*Individual-Level*				
Gender				
Female (vs. Male)	0.065 (0.030) *	0.240 (0.030) ***	0.319 (0.040) ***	0.466 (0.059) ***
Non-binary (vs. Male)	0.034 (0.095)	0.299 (0.102) **	0.458 (0.124) ***	0.468 (0.207) *
Questioning (vs. Male)	0.097 (0.145)	0.555 (0.101) ***	0.704 (0.136) ***	0.870 (0.140) ***
Parent Education	0.016 (0.011)	0.018 (0.011)	0.006 (0.014)	0.033 (0.021)
Generational Status				
Second Generation (vs. First)	−0.003 (0.051)	−0.052 (0.050)	0.074 (0.075)	0.175 (0.115)
Third Generation (vs. First)	−0.067 (0.055)	−0.132 (0.055) *	−0.015 (0.080)	0.154 (0.123)
Race/Ethnicity				
Black/African American (vs. White)	0.119 (0.061)	0.253 (0.057) ***	0.146 (0.078)	−0.005 (0.125)
East/Southeast Asian (vs. White)	0.060 (0.049)	−0.064 (0.053)	0.116 (0.079)	0.342 (0.114) **
Latiné (vs. White)	−0.033 (0.046)	0.053 (0.047)	0.135 (0.067) *	0.159 (0.096)
Multiethnic (vs. White)	−0.001 (0.053)	0.043 (0.054)	0.114 (0.069)	0.252 (0.097) **
Friendship Quality	0.188 (0.040) ***	0.325 (0.046) ***	0.158 (0.060) **	0.054 (0.091)
% White Friends	0.090 (0.068)	−0.048 (0.061)	−0.040 (0.092)	0.128 (0.119)

Note. * *p* < 0.05; ** *p* < 0.01; *** *p* < 0.001.

**Table 4:** Unstandardized coefficients from final multilevel models of proportion racial and ethnic minoritized friendships predicting 12th grade civic action, commitment to social change, awareness of inequality, and valuing diversity.

	Civic Action	Commitment to Social Change	Awareness of Inequality	Valuing Diversity
	*b*(SE)	*b*(SE)	*b*(SE)	*b*(SE)
Intercept	1.119 (0.143) ***	2.858 (0.161) ***	3.219 (0.318) ***	3.592 (0.208) ***
*School-Level*				
School Diversity	−0.019 (0.076)	−0.002 (0.075)	0.080 (0.139)	−0.032 (0.091)
% Free/Reduced Meals	0.001 (0.001)	0.001 (0.001)	−0.001 (0.002)	−0.002 (0.001) *
*Individual-Level*				
Gender				
Female (vs. Male)	0.065 (0.030) *	0.241 (0.030) ***	0.466 (0.059) ***	0.319 (0.040) ***
Non-binary (vs. Male)	0.033 (0.095)	0.297 (0.102) **	0.467 (0.207) *	0.455 (0.125) ***
Questioning (vs. Male)	0.097 (0.146)	0.556 (0.101) ***	0.869 (0.140) ***	0.706 (0.135) ***
Parent Education	0.017 (0.011)	0.017 (0.011)	0.034 (0.021)	0.006 (0.014)
Generational Status				
Second Generation (vs. First)	−0.003 (0.052)	−0.049 (0.051)	0.174 (0.115)	0.075 (0.074)
Third Generation (vs. First)	−0.063 (0.055)	−0.129 (0.055) *	0.154 (0.123)	−0.013 (0.080)
Race/Ethnicity				
Black/African American (vs. White)	0.107 (0.061)	0.250 (0.057) ***	−0.015 (0.125)	0.138 (0.077)
East/Southeast Asian (vs. White)	0.054 (0.049)	−0.065 (0.053)	0.333 (0.113) **	0.108 (0.079)
Latiné (vs. White)	−0.042 (0.046)	0.052 (0.047)	0.150 (0.095)	0.129 (0.066)
Multiethnic (vs. White)	−0.007 (0.053)	0.043 (0.053)	0.246 (0.097) *	0.109 (0.070)
Friendship Quality	0.190 (0.040) ***	0.323 (0.046) ***	0.057 (0.091)	0.159 (0.060) **
% Racial/Ethnic Minoritized Friends	−0.065 (0.057)	0.062 (0.059)	−0.104 (0.114)	0.061 (0.089)

Note. * *p* < 0.05; ** *p* < 0.01; *** *p* < 0.001.

## Data Availability

The datasets generated and/or analyzed for the current study are not publicly available but can be obtained from the corresponding author(s) upon reasonable request.

## References

[B1-behavsci-16-01501] Allport G. W. (1954). The nature of prejudice.

[B2-behavsci-16-01501] Árnadóttir K., Baysu G., Van Laar C., Phalet K., Tropp L. R., Sebben S., Ullrich J., Hässler T. (2024). How positive and negative intergroup contact jointly inform minority support for social change: The role of system-fairness beliefs. British Journal of Social Psychology.

[B3-behavsci-16-01501] Bentler P. M. (1990). Comparative fit indexes in structural models. Psychological Bulletin.

[B4-behavsci-16-01501] Berndt T. J. (2002). Friendship quality and social development. Current Directions in Psychological Science.

[B5-behavsci-16-01501] Bobek D., Zaff J., Li Y., Lerner R. M. (2009). Cognitive, emotional, and behavioral components of civic action: Towards an integrated measure of civic engagement. Journal of Applied Developmental Psychology, Foundations and Functions on Thriving in Adolescence.

[B6-behavsci-16-01501] Carter E. R., Brady S. T., Murdock-Perriera L. A., Gilbertson M. K., Ablorh T., Murphy M. C. (2019). The racial composition of students’ friendship networks predicts perceptions of injustice and involvement in collective action. Journal of Theoretical Social Psychology.

[B7-behavsci-16-01501] Cocco V. M., Vezzali L., Stathi S., Di Bernardo G. A., Dovidio J. F. (2024). Mobilizing or sedative effects? A narrative review of the association between intergroup contact and collective action among advantaged and disadvantaged groups. Personality and Social Psychology Review: An Official Journal of the Society for Personality and Social Psychology, Inc..

[B8-behavsci-16-01501] Cooperative Institutional Research Program (2011). CIRP construct technical report, 2011 appendix: Construct parameters.

[B9-behavsci-16-01501] Davies K., Tropp L. R., Aron A., Pettigrew T. F., Wright S. C. (2011). Cross-group friendships and intergroup attitudes: A meta-analytic review. Personality and Social Psychology Review.

[B10-behavsci-16-01501] Devine R. T., Grumley Traynor I., Ronchi L., Lecce S. (2024). Children in ethnically diverse classrooms and those with cross-ethnic friendships excel at understanding others’ minds. Child Development.

[B11-behavsci-16-01501] Diemer M. A., Pinedo A., Bañales J., Mathews C. J., Frisby M. B., Harris E. M., McAlister S. (2021). Recentering action in critical consciousness. Child Development Perspectives.

[B12-behavsci-16-01501] Dixon J., Elcheroth G., Kerr P., Drury J., Al Bzour M., Subašić E., Durrheim K., Green E. G. T. (2020). It’s not just “us” versus “them”: Moving beyond binary perspectives on intergroup processes. European Review of Social Psychology.

[B13-behavsci-16-01501] Dixon J., Tropp L. R., Durrheim K., Tredoux C. (2010). “Let them eat harmony”: Prejudice-reduction strategies and attitudes of historically disadvantaged groups. Current Directions in Psychological Science.

[B14-behavsci-16-01501] Dovidio J. F., Gaertner S. L., Saguy T. (2009). Commonality and the complexity of “we”: Social attitudes and social change. Personality and Social Psychology Review.

[B15-behavsci-16-01501] Flanagan C., Levine P. (2010). Civic engagement and the transition to adulthood. The Future of Children.

[B16-behavsci-16-01501] Glasford D. E., Dovidio J. F. (2011). E pluribus unum: Dual identity and minority group members’ motivation to engage in contact, as well as social change. Journal of Experimental Social Psychology.

[B17-behavsci-16-01501] Graham S. (2018). Race/ethnicity and social adjustment of adolescents: How (not if) school diversity matters. Educational Psychologist.

[B18-behavsci-16-01501] Graham S., Echols L. (2018). Race and ethnicity in peer relations research. Handbook of peer interactions, relationships, and groups.

[B19-behavsci-16-01501] Graham S., Munniksma A., Juvonen J. (2014). Psychosocial benefits of cross-ethnic friendships in urban middle schools. Child Development.

[B20-behavsci-16-01501] Hässler T., Ullrich J., Bernardino M., Shnabel N., Laar C. V., Valdenegro D., Sebben S., Tropp L. R., Visintin E. P., González R., Ditlmann R. K., Abrams D., Selvanathan H. P., Branković M., Wright S., Von Zimmermann J., Pasek M., Aydin A. L., Žeželj I., Ugarte L. M. (2020). A large-scale test of the link between intergroup contact and support for social change. Nature Human Behaviour.

[B21-behavsci-16-01501] Hässler T., Ullrich J., Sebben S., Shnabel N., Bernardino M., Valdenegro D., Van Laar C., González R., Visintin E. P., Tropp L. R., Ditlmann R. K., Abrams D., Aydin A. L., Pereira A., Selvanathan H. P., von Zimmermann J., Lantos N. A., Sainz M., Glenz A., Pistella J. (2022). Need satisfaction in intergroup contact: A multinational study of pathways toward social change. Journal of Personality and Social Psychology.

[B22-behavsci-16-01501] Hewstone M. (2015). Consequences of diversity for social cohesion and prejudice: The missing dimension of intergroup contact. Journal of Social Issues.

[B23-behavsci-16-01501] Jones J. M., Dovidio J. F. (2018). Change, challenge, and prospects for a diversity paradigm in social psychology. Social Issues and Policy Review.

[B24-behavsci-16-01501] Juvonen J., Kogachi K., Graham S. (2018). When and how do students benefit from ethnic diversity in middle school?. Child Development.

[B25-behavsci-16-01501] Kauff M., Asbrock F., Schmid K. (2021). Pro-diversity beliefs and intergroup relations. European Review of Social Psychology.

[B26-behavsci-16-01501] Leslie L. M., Bono J. E., Kim Y. S., Beaver G. R. (2020). On melting pots and salad bowls: A meta-analysis of the effects of identity-blind and identity-conscious diversity ideologies. Journal of Applied Psychology.

[B27-behavsci-16-01501] Levinson M., Sherrod L. R., Torney-Purta J., Flanagan C. A. (2010). The civic empowerment gap: Defining the problem and locating solutions. Handbook of research on civic engagement in youth.

[B28-behavsci-16-01501] Lorenzo K., Cham H., Yip T. (2024). Longitudinal associations between friendship ethnic/racial composition and ethnic/racial identity: The role of school ethnic/racial diversity. Journal of Youth and Adolescence.

[B29-behavsci-16-01501] MacCallum R. C., Kim C., Malarkey W. B., Kiecolt-Glaser J. K. (1997). Studying multivariate change using multilevel models and latent curve models. Multivariate Behavioral Research.

[B30-behavsci-16-01501] MacKinnon D. P., Lockwood C. M., Williams J. (2004). Confidence limits for the indirect effect: Distribution of the product and resampling methods. Multivariate Behavioral Research.

[B31-behavsci-16-01501] Northcutt Bohmert M., DeMaris A. (2015). Interracial friendship and the trajectory of prominority attitudes: Assessing intergroup contact theory. Group Processes & Intergroup Relations.

[B32-behavsci-16-01501] Pauker K., Carpinella C., Meyers C., Young D. M., Sanchez D. T. (2018). The role of diversity exposure in whites’ reduction in race essentialism over time. Social Psychological and Personality Science.

[B33-behavsci-16-01501] Pettigrew T. F., Tropp L. R. (2006). A meta-analytic test of intergroup contact theory. Journal of Personality and Social Psychology.

[B34-behavsci-16-01501] Pinedo A., Kubi G., Espinoza A. J., Gonzalez J., Diemer M. A. (2025). Ethnic studies and student development: Cultivating racially marginalized adolescents’ critical consciousness. Developmental Psychology.

[B35-behavsci-16-01501] Plummer J. A., Wray-Lake L., Alvis L., Metzger A., Syvertsen A. K. (2022). Assessing the link between adolescents’ awareness of inequality and civic engagement across time and racial/ethnic groups. Journal of Youth and Adolescence.

[B36-behavsci-16-01501] Ramos M. R., Li D., Bennett M. R., Mogra U., Massey D. S., Hewstone M. (2024). Variety is the spice of life: Diverse social networks are associated with social cohesion and well-being. Psychological Science.

[B37-behavsci-16-01501] Reimer N. K., Sengupta N. K. (2023). Meta-analysis of the “ironic” effects of intergroup contact. Journal of Personality and Social Psychology.

[B38-behavsci-16-01501] Rivas-Drake D., Umaña-Taylor A. J., Schaefer D. R., Medina M. (2017). Ethnic-racial identity and friendships in early adolescence. Child Development.

[B39-behavsci-16-01501] Rogers J., Kahne J., Ishimoto M., Kwako A., Stern S. C., Bingener C., Raphael L., Alkam S., Conde Y. (2022). Educating for a diverse democracy: The chilling role of political conflict in blue, purple, and red communities.

[B40-behavsci-16-01501] Ryu E. (2015). Multiple-group analysis approach to testing group difference in indirect effects. Behavior Research Methods.

[B41-behavsci-16-01501] Saguy T., Tausch N., Dovidio J. F., Pratto F. (2009). The irony of harmony: Intergroup contact can produce false expectations for equality. Psychological Science.

[B42-behavsci-16-01501] Sengupta N. K., Milojev P., Barlow F. K., Sibley C. G. (2015). Ingroup friendship and political mobilization among the disadvantaged. Cultural Diversity & Ethnic Minority Psychology.

[B43-behavsci-16-01501] Simpson E. H. (1949). Measurement of diversity. Nature.

[B44-behavsci-16-01501] Steiger J. H. (1998). A note on multiple sample extensions of the RMSEA fit index. Structural Equation Modeling: A Multidisciplinary Journal.

[B45-behavsci-16-01501] Terriquez V., Brenes T., Lopez A. (2018). Intersectionality as a multipurpose collective action frame: The case of the undocumented youth movement. Ethnicities.

[B46-behavsci-16-01501] Tropp L. R., Dehrone T. A. (2023). Prejudice reduction and social change: Dual goals to be pursued in tandem. The Oxford handbook of political psychology.

[B47-behavsci-16-01501] Tropp L. R., Hawi D. R., Van Laar C., Levin S. (2012). Cross-ethnic friendships, perceived discrimination, and their effects on ethnic activism over time: A longitudinal investigation of three ethnic minority groups. British Journal of Social Psychology.

[B48-behavsci-16-01501] Tropp L. R., Uluğ Ö. M., Uysal M. S. (2021). How intergroup contact and communication about group differences predict collective action intentions among advantaged groups. International Journal of Intercultural Relations.

[B49-behavsci-16-01501] Umaña-Taylor A. J., Lee R. M., Rivas-Drake D., Syed M., Seaton E., Quintana S. M., Cross W. E., Schwartz S. J., Yip T. (2014). Ethnic and racial identity during adolescence and into young adulthood: An integrated conceptualization. Child Development.

[B50-behavsci-16-01501] Wallrich L., West K., Rutland A. (2022). Valuing diversity: An undervalued mediator of intergroup contact. European Journal of Social Psychology.

[B51-behavsci-16-01501] Watts R. J., Flanagan C. (2007). Pushing the envelope on youth civic engagement: A developmental and liberation psychology perspective. Journal of Community Psychology.

[B52-behavsci-16-01501] Wegemer C. M. (2022). Service, activism, and friendships in high school: A longitudinal social network analysis of peer influence and critical beliefs. Journal of Youth and Adolescence.

[B53-behavsci-16-01501] Wilton L. S., Bell A. N., Vahradyan M., Kaiser C. R. (2020). Show don’t tell: Diversity dishonesty harms racial/ethnic minorities at work. Personality & Social Psychology Bulletin.

[B54-behavsci-16-01501] Wray-Lake L., Alvis L., Plummer J. A., Shubert J., Syvertsen A. K. (2023). Adolescents’ developing awareness of inequality: Racial and ethnic differences in trajectories. Child Development.

